# A systematic review of natural killer cells profile and cytotoxic function in myalgic encephalomyelitis/chronic fatigue syndrome

**DOI:** 10.1186/s13643-019-1202-6

**Published:** 2019-11-14

**Authors:** Natalie Eaton-Fitch, Stanley du Preez, Hélène Cabanas, Donald Staines, Sonya Marshall-Gradisnik

**Affiliations:** 1 National Centre for Neuroimmunology and Emerging Diseases, Menzies Health Institute, Griffith University, Gold Coast, Australia; 2 School of Medical Science, Griffith University, Gold Coast, Australia; 3 School of Medicine, Griffith University, Gold Coast, Australia

**Keywords:** Chronic fatigue syndrome, Myalgic encephalomyelitis, Natural killer cells, Cytotoxicity

## Abstract

**Background:**

Compromised natural killer (NK) cell cytotoxic function is a well-documented and consistent feature of myalgic encephalomyelitis/chronic fatigue syndrome (ME/CFS). Other outcomes evaluated in NK cells of ME/CFS patients, however, remain equivocal. The aim of this study was to conduct a systematic review of the literature regarding NK cell phenotype, receptor expression, cytokine production and cytotoxicity in ME/CFS patients and determine the appropriateness as a model for ME/CFS.

**Methods:**

Medline (EBSCOHost), Scopus, EMBASE and PubMed databases were systematically searched to source relevant papers published between 1994 and March 2018. This review included studies examining NK cells’ features in ME/CFS patients compared with HC following administration of specific inclusion and exclusion criteria. Secondary outcomes included genetic analysis in isolated NK cells or quality of life assessment. Quality assessment was completed using the Downs and Black checklist in addition to The Joanna Briggs Institute checklist.

**Results:**

Seventeen eligible publications were included in this review. All studies were observational case control studies. Of these, 11 investigated NK cell cytotoxicity, 14 investigated NK cell phenotype and receptor profiles, three examined NK cell cytokine production, six investigated NK cell lytic protein levels and four investigated NK cell degranulation. Impaired NK cell cytotoxicity remained the most consistent immunological report across all publications. Other outcomes investigated differed between studies.

**Conclusion:**

A consistent finding among all papers included in this review was impaired NK cell cytotoxicity, suggesting that it is a reliable and appropriate cellular model for continued research in ME/CFS patients. Aberrations in NK cell lytic protein levels were also reported. Although additional research is recommended, current research provides a foundation for subsequent investigations. It is possible that NK cell abnormalities can be used to characterise a subset of ME/CFS due to the heterogeneity of both the illness itself and findings between studies investigating specific features of NK function.

## Background

Myalgic encephalomyelitis (ME), also referred to as chronic fatigue syndrome (CFS), is a clinically defined condition characterised by profound dysregulation of the central nervous system and immune system [1, 2], endocrine dysfunction [3], and impaired cellular energy metabolism and ion transport [4, 5]. The global prevalence of ME/CFS is reported to range from 0.2 to 6.3%; however, this is difficult to accurately determine due to the absence of a diagnostic test [6]. Without a biological marker, diagnosis currently relies on the exclusion of all other possible fatigue-related illnesses and identification of ME/CFS cases using various symptom-based criteria [7–9].

In 1994, the Centers for Disease Control and Prevention published the Fukuda Criteria to evaluate and classify ME/CFS patients and provide a basis for diagnosis [8]. A case of ME/CFS is defined under these criteria by the presence of unexplainable chronic fatigue that is not alleviated by rest. At least four additional concurrent symptoms including sore throat, tender lymph nodes, muscle and/or joint pain, impaired cognition and sleep disturbances are necessary for diagnosis. Revised protocols birthed the Canadian Consensus Criteria (CCC) (2003) and the International Consensus Criteria (ICC) (2011) [7, 9]. Post-exertional neuroimmune exhaustion accompanied by numerous neurological, autonomic and neuroendocrine manifestations are notable elements of these revised definitions necessary to formally diagnose a case of ME/CFS.

The aetiology of ME/CFS remains elusive. The involvement of the immune system is supported by the consistent observation of features representative of a ‘flu-like’ illness in addition to reports of disturbed cytokine profiles [10–13], decreased natural killer (NK) cell activity and reduced T lymphocyte response [1, 5, 14–16]. Decreased NK cell activity is considered the most consistent immunological observation in ME/CFS patients [1, 7, 15–19]. Several studies have reported significantly decreased NK cell function in ME/CFS patients compared with healthy controls (HC) [1, 2, 14, 19–28]. These studies have demonstrated variations in NK cell phenotype and regulatory receptors, significantly reduced cytolytic proteins, impaired mitogen-activated protein kinases (MAPK) phosphorylation, increased expression of degranulation markers and impaired calcium (Ca^2+^) mobilisation.

NK cells are large granular lymphocytes of the innate immune system with natural cytotoxicity against tumour cells and virus-infected cells independent of prior sensitisation and in a non-MHC restricted manner [29]. NK cells have a protective role in various inflammatory conditions through immune cell activation, cytokine production and direct cytotoxicity [29]. In human peripheral blood, NK cell sub-populations are defined by their expression of cell-surface molecules, namely CD56 and CD16, which can distinguish cells into the following subsets: CD56^Bright^CD16^−^, CD56^Bright^CD16^Dim-^, CD56^Dim^CD16^−^, CD56^Dim^CD16^Bright^, CD56^−^CD16^Bright^ [30]. CD56^Dim^CD16^Bright^ NK cells represent at least 90% of all peripheral NK cells and display significantly higher cytolytic capacity against infected or malignant target cells as this sub-population contains more cytolytic proteins and form more conjugates with target cells [31, 32]. CD56^Bright^ NK cells are potent cytokine producers. The major cytokines produced include interferon-γ (IFN-γ), tumour necrosis factor-α (TNF-α), granulocyte-macrophage colony-stimulating factor, interleukin (IL)-10 and IL-13 [30].

NK cell function relies on the rise of intracellular Ca^2+^ concentrations [33]. Several steps during cytotoxicity are Ca^2+^-dependent including lytic granule polarisation, immune synapse formation and exocytosis of cytolytic proteins [33]. During NK cell activation, the interaction between NK cells and target cells initiates intracellular signals through the MAPK phosphorylation cascade [34]. Downstream phosphorylation of MAPK is responsible for the polarisation and release of cytotoxic granules, otherwise referred to as degranulation. Degranulation marker CD107a is expressed extracellularly following NK cell activation and is used to detect functional activity of NK cells [35]. NK cell cytotoxicity involves the exocytosis of lytic proteins, predominantly perforin, granzyme A and granzyme B, and concludes with apoptosis of the target cell. Perforin, a membrane-disrupting glycoprotein, creates a pore to facilitate the influx of granzyme proteases [36]. Granzyme B possesses the strongest apoptotic activity owing to its ability to rapidly cleave and activate procaspases, ultimately leading to deoxyribonucleic acid (DNA) fragmentation and subsequent cell death [37]. Conversely, granzyme A is a slow-acting activator of apoptosis [38].

Previous investigators have reported equivocal differences in NK cell phenotype, cytokine production and cytotoxicity. However, it is believed that ME/CFS severity or a subset of disease is associated with specific NK cell sub-populations and functional profiles. Using NK cells as vectors for research and diagnostic approaches for ME/CFS is supported by a growing body of evidence, which will be examined in this review. Specifically, NK cell phenotype, receptor expression, cytokine production and cytotoxicity in ME/CFS will be the focus of this review.

## Method

### Literature search

This review was performed according to PRISMA (Preferred Reporting Items for Systematic Reviews and Meta-analyses) guidelines. PubMed, Scopus, EMBASE and Medline (EBSCOHost) databases were searched. The following full-text terms were searched: ‘chronic fatigue syndrome’ OR ‘myalgic encephalomyelitis’ OR ‘ME/CFS’ AND natural killer cell*. Medical subject headings (MeSH) terms were used for chronic fatigue syndrome/myalgic encephalomyelitis (including systematic exertion intolerance disease) and natural killer cells. Boolean operators ‘OR’ was used to combine all expressions of cases including abbreviation while ‘AND’ was used to include NK cells in conjunction with ME/CFS in the search. Proximity operators were not used during the literature search. Two literature searches were completed in this systematic review on separate occasions by two authors and using the same method. Reference list checking and citation searching was completed, and no additional papers were selected. Searching for unpublished literature was not performed. The primary search completed by the first author (NEF) was on 31st of May 2018 and the final search concluded by another author was on 21st of August 2018 (by HC). No additional papers were identified in the final search or through alternative search databases such as Griffith University institute library or Google Scholar.

### Inclusion and exclusion criteria

This systematic review was designed to include observational studies using quantitative methods to compared NK cell profiles and cytotoxic activity in ME/CFS patients compared with HC. Titles and abstracts were screened according to the following criteria by two authors (NEF and HC): (i) all studies reported on NK cell cytotoxicity, NK cell phenotype or receptor profiles, MAPK phosphorylation, degranulation or lytic proteins in ME/CFS patients compared with HC as their primary outcome; (ii) studies were published between 1994 and 2018 to exclude non-Fukuda-based case definitions; (iii) ME/CFS diagnosis fulfilled either Fukuda, CCC or ICC; (iv) human studies in adults age 18 years and above; (v) free full text publications available through institutional access; and (vi) based upon original research.

Studies were excluded if only one out of the two keywords were present in the title or abstract. Studies were excluded if the ME/CFS cohort was compared with another patient group (e.g. fibromyalgia (FM), multiple sclerosis (MS), chronic fatigue) and not compared with HC. Studies were excluded if pharmacological, exercise or sleep interventions were used. Secondary outcomes evaluated include genetic investigations and quality of life (QoL).

### Screening of the articles

All papers obtained from the search were imported to Zotero for storage and subsequent screening. To remove the potential for selection bias, two authors also independently completed the screening of papers. The inclusion and exclusion criteria mentioned above were used for publication selection. After review of abstracts and titles by two authors, full texts were also screened. Publications that met the criteria for inclusion were finally reviewed by another author (SMG) and underwent data collection.

### Selection of studies and data extraction

Following screening of titles and abstracts, eligible studies were analysed and the following details were extracted and summarised in Tables 1, 2 and 3: (i) author, (ii) year, (iii) country, (iv) study design, (v) sample type (i.e. ME/CFS or HC), (vi) sample size, (vii) outcome(s) and (viii) statistical results. Two authors independently assessed full-text articles for suitability for inclusion in this review.

### Search strategy validation

The search strategy used in this systematic review was validated by an independent party on Wednesday 10th April 2019. No additional papers were found during validation and no papers were excluded.

### Quality assessment

Studies were evaluated for quality and bias (performed by NEF and SDP) using the Joanna Briggs Institute (JBI) Checklist for case controls [39]. Additionally, the Downs and Black checklist was also included for items asking further information pertaining to clear description of outcomes and findings, reported probability outcomes, recruitment details and participant representation of populations [40, 41]. Items 3, 4, 8, 9, 13, 14, 15, 17–19, 23–27 of the Downs and Black checklist were excluded due to their specificity for interventional studies and overlap with the JBI checklist.

## Results

**Fig. 1:**
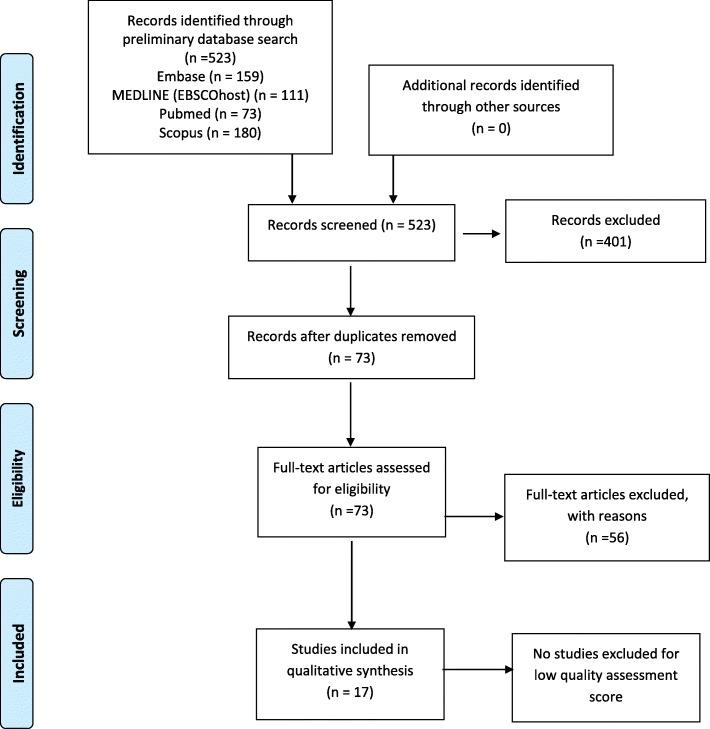
PRISMA flow diagram of literature search displaying selection and exclusion of publications

A total of 523 papers were identified from Medline (EBSCOhost) (111), Embase (159), PubMed (73) and Scopus (180). Papers were screened according to the aforementioned inclusion and exclusion criteria. Figure 1 summarises the results of the literature search using PRISMA.

### Overview of papers

The PRISMA flow diagram including information of papers screened, excluded and included is displayed in Fig. 1. The characteristics and primary outcomes of the 17 papers included in this review are summarised in Tables 1 and 2. All papers in this review were observational case control studies that examined NK cells in ME/CFS patients compared with HC. No potentially relevant papers were excluded from this review dependent on availability. At the end of the search and screening of the papers, authors reported no discrepancies.

**Table 1:** Summary of study and participant characteristics

Paper details						Sample size	
Author	Year	Study design	Diagnostic criteria	Country	Sample	ME/CFS (female%)[years]	HC (female%)[years]
Brenu et al. [42]	2010	Case Control	Fukuda	Australia	NK cells	10	10
Brenu et al. [2]	2011	Case Control	Fukuda	Australia	NK cells	95 (70.5%) [46.47 ± 11.7]	50 (57.7%) [41.9 ± 9.6]
Brenu et al. [43]	2012	Case Control	Fukuda	Australia	NK cells	65 (75.4%) [47.2 ± 11.5]	21 (66.7%) [45.2 ± 9.3]
Brenu et al. [14]	2013	Case Control	Fukuda	Australia	PBMCs	30 [51.15 ± 1.92]	25 [50.42 ± 1.76]
Curriu et al. [48]	2013	Case Control	Fukuda	Spain	PBMCs	22 (73%) [44]	30 (55%) [38]
Fletcher et al. [45]	2010	Case Control	Fukuda	USA	PBMCs	176 (83%) [44]	230 (86%) [41]
Hardcastle et al. [46]	2015	Case Control	Fukuda	Australia	PBMCs	Severe *n* = 12 (83%) [41.27 ± 10.05]	18 (72%)[41.94 ± 10.76]
						Moderate *n* = 12 (67%) [44.73 ± 12.9]	
Hardcastle et al. [46]	2015	Case Control	Fukuda	Australia	PBMCs, NK cells	Severe *n* = 12 (83.3%) [41.25 ± 2.77]	18 (66.7%) [40.39 ± 2.65]
						Moderate *n* = 15 (73.3%) [45.93 ± 2.96]	
Huth et al. [21]	2014	Case Control	Fukuda	Australia	NK cells	29 [48.28 + 2.63]	27 [49.15 + 2.51]
Huth et al. [16]	2016	Case Control	Fukuda	Australia	PBMCs	14 [53.5 ± 2.17]	11 [48.82 ± 3.46]
Maher et al. [20]	2005	Case Control	Fukuda	USA	Whole blood	30 (83.3%) [46 ± 10]	19 [43 ± 10]
Marshall-Gradisnik et al. [44]	2016	Case Control	Fukuda	Australia	NK cells	39 (71.8%) [51.69 ± 2]	30 (56.7%) [47.6 ± 2.39]
Nguyen et al. [49]	2016	Case Control	Fukuda	Australia	PBMCs	17 (82%) [48.68 ± 1.06]	19 (68%) [46.48 ± 1.22]
Nguyen et al. [5]	2017	Case Control	Fukuda	Australia	NK cells	25 (68%) [48.82 ± 9.84]	15 (70.6%) [39.2 ± 12.12]
Rivas et al. [51]	2018	Case Control	CCC	Spain	Whole blood	76 (82.9%) [49.78]	73 (82.2%) [48.71]
Stewart et al. [50]	2003	Case Control	Fukuda	USA	Whole blood	90 (69%) [36.7 ± 7.3]	50 (76%) [35.7 ± 9.2]
Theorell et al. [47]	2017	Case Control	CCC	Sweden, Norway	PBMCs	24 (75%) [44]^a^	28 (71%) [44]^a^
						24 (79%) [30]^b^	24 (79%) [30]^b^

*ME*/*CFS* myalgic encephalomyelitis/chronic fatigue syndrome, *HC* healthy control, *USA* United States of America, *NK* natural killer, *PBMC* peripheral blood mononuclear cells

^a^Location: Stockholm

^b^Location: Oslo

**Table 2:** Summary of primary outcome results

Author	Year	Assessed	Method	Increased in ME/CFS vs. HC (*p* value)	Decreased in ME/CFS vs. HC (*p* value)	No significance
Brenu et al. [42]	2010	NK cell phenotypes	Flow cytometry		CD56^Bright^ (*p* < 0.05)	CD56^Dim^
		NK cell cytotoxicity			K562% lysis (*p* < 0.05)	
Brenu et al. [2]	2011	NK cell phenotypes	Flow cytometry		CD56^Bright^ (*p* < 0.001)	CD56^Dim^
		NK cell cytotoxicity			K562% lysis (*p* < 0.05)	
Brenu et al. [43]	2012	NK cell phenotypes	Flow cytometry		CD56^Bright^ (*p* = 0.02)^a^	CD56^Dim^
		NK cell cytotoxicity			K562% lysis (p < 0.05)^a^	
Brenu et al. [14]	2013	NK cell phenotypes	Flow cytometry			CD56^Bright^, CD56^Dim^ KIR, NKG2D
		NK cell cytotoxicity			K562% (*p* < 0.05)	
		NK cell degranulation		CD107a (*p* < 0.05)		
		NK cell lytic proteins			Granzyme B (*p* < 0.05)	Perforin, Granzyme A
		NK cell cytokine production		IFN-y (*p* < 0.05)		
Curriu et al. [48]	2013	NK cell phenotypes	Flow cytometry	CD69 (*p* = 0.0004, *p* < 0.01)^b^ NKp46 (*p* < 0.001), (*p* = 0.0023)^b^	CD57 (*p* = 0.052) (MFI) CD25CD56^Bright^ (*p* = 0.0012) CD25CD56^Dim^ (*p* = 0.0494)	CD56^Bright^, CD56^Dim^ CD57 (*p* = 0.1942)
		NK cell cytotoxicity				K562% lysis
Fletcher et al. [45]	2010	NK cell cytotoxicity	^51^Cr release		^51^Cr (*p* < 0.000)	
Hardcastle et al. [46]	2015	NK cell phenotype	Flow cytometry	KIR3DL1/DL2 (*p* < 0.004)^a^ KIR2DL1 (*p* = 0.011)^a^ KIR2DL2/DL3 (0.049)^a^ KIR2DS4 (0.023)^a^ CD18^+^CD11c^−^ (*p* = 0.036) CD18^+^CD2^−^ (*p* = 0.009) SLAM (*p* = 0.046)	CD56^Bright^NKG2D (*p* = 0.014) KIR2DL2/DL3 (*p* = 0.045)^a^ NKp46 (*p* = 0.021)	
		NK cell lytic proteins				Perforin, granzyme A, granzyme B
Huth et al. [21]	2014	NK cell phenotype	Flow cytometry		CD56^Bright^CD2^+^CD18^+^ (*p* < 0.01) CD56^Dim^CD18^+^CD11a^+^CD11c^+^ (*p* < 0.05)	CD56^Dim^CD2^+^CD18^+^ CD18^+^CD11a^+^CD11b^+^CD11c^+^
		NK cell degranulation				CD107a/b
		NK cell lytic proteins			CD56^Dim^ Granzyme B (*p* < 0.05)	Granzyme A, Perforin
Huth et al. [16]	2016	NK cell MAPK phosphorylation	Flow cytometry	MEK1/2 (*p* < 0.05) P38 (*p* < 0.05)	ERK1/2 (*p* < 0.05)	MEK1/2 P38
		NK cell cytotoxicity			K562% lysis	
		NK cell degranulation				CD107a/b
		NK cell lytic proteins				Perforin, Granzyme A, granzyme B
		NK cell phenotype				CD56^Bright^, CD56^Dim^
		NK cell cytokines				IFN-γ TNF-α GM-CSF
Maher et al. [20]	2005	NK cell phenotype	Flow cytometry		CD56^+^CD3^−^ (*p* = 0.04)	
		NK cell cytotoxicity	^51^Cr release		^51^Cr (*p* = 0.001)	
		NK cell lytic proteins	Flow cytometry		Perforin (*p* = 0.01)	
Marshall-Gradisnik et al. [44]	2016	NK cell cytotoxicity	Flow cytometry		K562% lysis (*p* < 0.05).	
Nguyen et al. [49]	2016	NK cell phenotype	Flow cytometry		TRPM3^+^ expression in CD56^Bright^ (*p* < 0.05)	TRPM3^+^ expression in CD56^Dim^
		NK cell Ca^2+^ influx			CD56^Bright^ (*p* < 0.05)^c^	
Nguyen et al. [5]	2017	NK cell phenotype	Flow cytometry	CD56^Dim^TRPM3^+^ (*p* < 0.01)^c^	TRPM3^+^ expression in CD56^Bright^ (*p* < 0.05) CD69 (*p* < 0.05)^c^	CD69
		NK cell degranulation				CD107a
		NK cell Ca^2+^ influx		CD56^Bright^ (*p* < 0.05)^c^		CD56^Dim^
		NK cell cytotoxicity		K562% lysis (*p* < 0.05)^c^		
Rivas et al. [51]	2018	NK cell phenotype	Flow cytometry	CD69 (*p* = 0.0011) CD56^Bright^ (*p* = 0.0075)	NKG2C (*p* < 0.0001)	
Stewart et al. [50]	2003	NK cell phenotype	Flow cytometry		CD56^Bright^CD8^+^ (*p* = 0.04) CD56^Bright^CD8^−^ (*p* = 0.05)^d^	
Theorell et al. [47]	2017	NK cell phenotype	Flow cytometry	CD56^Dim^ HLA-DR (*p* = 0.002)^d^		
		NK cell cytotoxicity	^51^Cr release			K562% lysis IFN-γ inhibition (*p* = 0.004, 0.009)^c^
		NK cell lytic proteins	Flow cytometry			Perforin, Granzyme A/B
		NK cell cytokines		CD56^Dim^ IFNγ (p = 0.009)^c^		
		NK cell degranulation				CD107a

*ME* myalgic encephalomyelitis, *CFS* chronic fatigue syndrome, *HC* healthy control, *NK* natural killer, *Ca*^2*+*^ calcium, *NS* no significance, *TNF* tumour necrosis factor, *IFN* interferon, *MFI* mean fluorescence intensity

^a^Significance dependent on longitudinal data

^b^Significance is NK cell phenotype dependent

^c^Significance reported post pharmacological stimulation

^d^Significance dependent on geographical location

### Participant and study characteristics

The mean number of participants across all papers was 89. Specifically, the mean number of ME/CFS patients was 48 and 41 for HC.

Six studies used isolated NK cells [2, 5, 21, 42–44], seven used PBMCs [23, 25, 45–49], three used whole blood [20, 50, 51] and one used PBMCs as well as isolated NK cells [17]. Primary outcomes included NK cell cytotoxicity, NK cell immunophenotyping and receptor/channel expression, intracellular lytic protein stores and degranulation. NK cell phenotyping was completed in 11 studies [2, 17, 20, 21, 23, 25, 42, 43, 47, 48, 51]; 11 examined NK cell cytotoxicity [2, 5, 20, 23, 25, 42–45, 47]; five examined NK cell degranulation [5, 21, 23, 25, 47]; six examined NK cell lytic proteins [20, 21, 23, 25, 46, 47]; three examined cytokine production [23, 25, 47]; six examined NK cell receptors and markers [5, 17, 25, 45, 47, 48]; one examined NK cell MAPK phosphorylation [23]; and two examined NK Cell Ca^2+^ influx [5, 49].

Other outcomes included genotyping and polymorphism analysis in addition to QoL scores. Three of the studies reported genotyping analysis [2, 44, 47]; one used the short form 36 health survey (SF-36) [51]; one used the Fatigue Severity Scale (FSS) [17]; two employed Dr. Bell’s Disability Scale [17, 46]; two used the Karnofsky Performance Scale [17, 46]; and one applied the FibroFatigue Scale [17].

### Literature reporting NK cell cytotoxic function in ME/CFS patients

Studies that analysed NK cell cytotoxicity are summarised in Table 2. Of these papers, seven reported significant reduction in cytotoxic activity in ME/CFS patients compared with HC [2, 20, 25, 42–45]. Additionally, one study reported decreased inhibition of NK cell cytotoxicity following exposure to 0.1 μM (*p* = 0.004) and 0.01 μM (*p* = 0.009) of adrenaline, however did not reach significance [47]. One of these studies analysed NK cell cytotoxicity over 12 months [45]. This paper reported significant reductions in NK cell cytotoxicity in ME/CFS patients compared with HC at 0 month, 6 months and 12 months (*p* < 0.05). Two of these seven papers used the Chromium (^51^Cr) release assay to determine the percentage of NK cell target cell death [20, 45], while the remaining five used flow cytometry [2, 25, 42–44].

Of the 11 publications examining NK cell cytotoxicity, three reported no significant changes after stimulation with a target cell line alone [5, 47, 48] (Table 2); however, methods were not consistent which may not reflect the validity of NK cell cytotoxicity results.

### Literature reporting changes in NK cell Immunophenotype

Of the 17 publications included in this review, 14 investigated NK cell phenotypes and receptor expression (Table 2) [2, 5, 17, 20, 21, 23, 25, 42, 43, 47–51]. Four studies reported a significant decrease in CD56^Bright^ NK cell subset in ME/CFS patients compared with HC [2, 42, 43, 50]. One paper reported a significant reduction in CD56^Bright^ NK cells after 6 months in the ME/CFS patient group [43]. Stewart et al. reported a significant decrease in CD56^Bright^CD8^+/−^ NK cells [50], while an increase in CD56^Bright^ NK cells was reported in another paper [51]. Of the 14 papers reporting on NK cell phenotype and surface marker expression, five lacked significance for the CD56^Dim^ NK cell subset between ME/CFS patients and HC [2, 17, 23, 42, 43], while three reported no significance in CD56^Bright^ NK cells between groups [23, 25, 48].

One study reported increased MHC Class II receptor HLA-DR on CD56^Dim^ NK cells [47]. Two studies investigated transient receptor potential (TRP) TRPM (melastatin) 3 channel expression on NK cells, with both reporting a significant decrease in CD56^Bright^TRPM3^+^ expression [5, 49], whereas a significant increase was reported for CD56^Dim^TRPM^+^ in one paper [5]. Note that TRPM3 expression on CD56^Bright^ NK cells was significant without stimulants, whereas CD56^Dim^ TRPM3 expression was significant following Pregnenolone sulfate and Ionomycin stimulation [5]. Two papers examined CD2, CD11 and CD18 expression on NK cells [17, 21]. One study reported a significant increase in CD18^+^CD11c^−^ and CD18^+^CD2^−^ NK cells in ME/CFS patients compared with HC [17], while another reported a significant decrease in CD56^Bright^CD2^+^CD18^+^ and CD56^Dim^CD18^+^CD11a^+^CD11c^+^ NK cells [21].

Five papers examined NK cell expression of killer-cell immunoglobulin-like receptors (KIRs), activating receptors or signalling lymphocytic activation molecule (SLAM) receptors [17, 21, 46, 48, 51]. Huth et al. reported no significant differences between ME/CFS patients and HC [21]. Conversely, one paper reported significantly increased SLAM receptors [17] and another reported significant increases in selected KIRs and a decrease in NK receptor group 2 member D (NKG2D) and KIR2DL2/DL3 [46]. One investigation reported significantly increased NKp46 [48], whereas another reported significantly decreased NKp46 [17]. The remaining paper reported a significant decrease in NKG2C in ME/CFS patients compared with HC [51].

Moreover, two papers reported significantly increased expression of activation marker CD69 [48, 51] in ME/CFS patients compared with HC. By contrast, one paper reported significantly decreased CD69 expression in ME/CFS patients compared with HC following pharmacological and target cell stimulation; however, the significance was not reported at baseline [5].

### Literature reporting changes in NK cell cytokine production

Three papers examined NK cell-dependent cytokine production in ME/CFS patients compared with HC [23, 25, 47] (Table 2). Two reported significantly increased IFN-γ production [25, 47], while one reported no significant differences in IFN-γ, TNFα and GM-CSF between control and patient groups [23].

### Literature reporting changes in NK cell lytic proteins

Six publications examined lytic proteins in NK cells of ME/CFS patients compared with HC [20, 21, 23, 25, 45, 47] (Table 2). A significant reduction in Granzyme B was reported in two papers [21, 25]. One publication reported a significant reduction in perforin [20]. No significant differences were reported in four publications for perforin and granzyme A [21, 23, 25, 45] and three reported no significant changes in Granzyme B [23, 45, 47].

### Literature reporting changes in degranulation

Four of the included papers investigated the NK cell degranulation markers CD107a and CD107b [5, 21, 23, 25] (Table 2). One paper reported a significant increase in CD107a [25]. Conversely, three publications lacked significance for these degranulation markers [5, 21, 23].

### Literature reporting secondary outcomes

Three of the 17 papers included genotype analysis in ME/CFS patients [2, 44, 47] (Table 3). One study used quantitative reverse transcription polymerase chain reaction (qRT-PCR) to measure gene expression [2]. Brenu et al. reported a significant reduction in mRNA coding for granzyme A, granzyme K, perforin, and IFN-γ. Additionally, one study examined polymorphisms in PRF1, the gene coding perforin, however did not report any significant difference [47]. Lastly, one paper examined 678 single-nucleotide polymorphisms (SNPs) in ME/CFS patients; of these, 11 were associated with TRPC (Canonical) 4, TRPC2, TRPM3 and TRPM8; and 14 SNPs were associated with nicotinic and muscarinic acetylcholine receptors (AChR) [44].

**Table 3:** Summary of genotyping secondary outcomes

Author	Year	Assessed	Method	Significance	NS
Brenu et al. [2]	2011	NK cell lytic proteins	qRT-PCR	Granzyme A (*p* < 0.05) Graznyme K (*p* < 0.05) IFN-γ (*p* < 0.05)	
Marshall-Gradisnik et al. [44]	2016	NK cell ion channel SNP		TRPC4 (*p* < 0.05) TRPC2 (*p* < 0.05) TRPM3 (*p* < 0.05) TRPM8 (*p* < 0.05)	
		AChR receptors		CHRNA3 (*p* < 0.05) CHRNA2 (*p* < 0.05) CHRNB4 (*p* < 0.05) CHRNA5 (*p* < 0.05) CHRNE (*p* < 0.05)	
Theorell et al. [47]	2017	Perforin (PRF1)	PCR		*PRF1*

*NK* natural killer, *NS* no significance, *qRT*-*PCR* qualitative reverse transcription polymerase change reaction, *SNP* single-nucleotide polymorphism, *PCR* polymerase chain reaction, *AChR* acetylcholine receptors, *TRPC* transient receptor potential canonical, *TRPM* transient receptor potential melastatin

Three papers examined correlation between NK cell features and patient severity or QoL [17, 51, 52]. Hardcastle et al. reported a negative correlation between Dr. Bell’s Disability Scale and the Karnofsky Performance Scale (KPS) with CD56^Dim^ NK cells with CD18^+^CD11c^−^ in ME/CFS patients [17]. One study used the SF-36 to examine patient severity and QoL in association with NK cell phenotypes [51]. This publication reported a negative trend between fatigue and pain scores with NKG2C expression in ME/CFS patients where this was not observed in HC.

### Quality assessment of papers

All papers were assessed for quality and bias by two authors using both the Downs and Black checklist and the JBI checklist for case-control studies (Additional file 1: Table S1). The JBI checklist was used due to its specificity with case-control studies. All 17 papers met the JBI criteria for (i) criteria used for cases and controls; (ii) outcomes assessed in standard, valid and reliable way; and (iii) appropriate statistical analysis was used. All 17 papers met the Downs and Black criteria for (i) aims and objectives clearly described, (ii) main outcomes clearly described, (iii) main findings clearly described, (iv) provides estimates of random variability, (v) studies were without data dredging and (vi) main outcomes measured were accurate. Twelve of the 17 papers met the Downs and Black checklist for actual probability values [5, 17, 20, 25, 45–48, 50, 51]. No papers were representative of the entire population from which they were recruited. One publication commented on recruitment time period [43].

All papers provided an internationally accepted case definition for ME/CFS patients and stated appropriate exclusion criteria. Minimal information was provided for the inclusion of HC for the papers included in this systematic review. All papers, excluding Huth et al. (2014) and Nguyen et al. (2016) [24, 52], appropriately matched HC and ME/CFS participants. No papers commented on controlling for confounding variables.

## Discussion

The aim of this systematic review was to summarise and examine the evidence available on NK cells in ME/CFS patients. NK cell cytotoxicity, immunophenotype, degranulation, lytic proteins and cytokine production were analysed. Seventeen studies met the inclusion criteria for this review and demonstrated a consistent loss in NK cell cytotoxicity; however, reports regarding the other mentioned outcomes were inconsistent.

### Study characteristics

A significant limitation of 15 of the 17 studies was the use of the Fukuda criteria. In comparison to other definitions such as the CCC and ICC, the Fukuda definition is considered broad and can predispose to the misdiagnosis of ME/CFS as the defining symptoms are not specific or limited to ME/CFS. The release of the Fukuda definition was intended to guide ME/CFS research in adult populations [53]. For this reason, the Fukuda definition is the most widely used definition in ME/CFS research and clinical evaluation of patients. An important feature of the CCC and ICC definitions is the requirement of post-exertional malaise and neuroimmune exhaustion, respectively. A review by Brurberg and colleagues examined case definitions employed by 38 studies and reported that no empirical data indicated that any case definition specifically identified ME/CFS patients as having a neuroimmunological condition [54], suggesting that these revised definitions are not vastly superior to the original Fukuda criteria in discerning cases of ME/CFS. Regardless, all these diagnostic criteria require the exclusion of any active or previous medical conditions that may explain for the presence of symptoms.

A consistent laboratory method was the use of flow cytometry to analyse fluorescence of target proteins on NK cells. Flow cytometry is considered a gold standard technique when measuring cell function, expression of surface markers, cytokine and signalling proteins and discriminating between apoptotic and viable cells. Three papers used the ^51^Cr release assay to measure NK cell cytotoxic activity [20, 45, 47]. Comparison studies have shown flow cytometric methods to be more sensitive and obtain higher target cell lysis values than the ^51^Cr release assay [55, 56]. However, the use of both flow cytometry and ^51^Cr release assay in different papers included in this review yielded consistent results of reduced NK cell cytotoxicity.

### Natural killer cells in ME/CFS

The findings generated from investigations into NK cell phenotypes in ME/CFS patients vary. A significant reduction in CD56^Bright^ NK cell subset was a consistent finding across four of the 11 papers reporting on NK cell phenotype. A recent study included in this systematic review, which consisted of 76 ME/CFS patients defined in accordance with the CCC matched with 73 HC, reported that CD56^Bright^ NK cell subset was significantly higher in ME/CFS patients [51]. This report is not consistent with earlier reports using low sample sizes that employed the Fukuda criteria. Hence, it emphasises the need for consistent case definitions and similar sample sizes to be used to facilitate comparison and may suggest anomalies in NK cell phenotypic profiles in ME/CFS patients or a subset of patients. For example, reduced CD56^Bright^ NK cells are often observed in patients with juvenile rheumatoid arthritis [30]. Pridgen et al. reported that NK cell subsets in peripheral blood lacked significant differences to samples from healthy donors; however, synovial fluid of adult rheumatoid arthritis patients almost exclusively contained CD56^Bright^ NK cells [57]. Thus, any inconsistencies in results presented in this review may be explained by the distribution of NK cells in different tissues or may be representative of a subset of patients. Moreover, a limitation of these studies is that some authors investigated different CD56^Bright^CD16^−^ or CD56^Bright^CD16^Dim^ subsets, rendering it difficult to make comparisons or draw conclusions. A review by Poli et al. suggested research techniques be harmonised and both sub-populations be grouped together as CD56^Bright^ NK cells [30], which is supported by the current review.

The reactivity of NK cells is determined by the balance of activating and inhibitory receptors, including, but not limited to, KIRs and natural cytotoxicity receptors (NCRs). NCRs including NKp46, NKp30 and NKp44 are involved in virally infected and tumour cell elimination [58]. There are ambiguous and limited reports on NK cell receptor expression in ME/CFS patients. An Australian investigation reported decreased NKp46 expression in ME/CFS patients [17], whereas one publication included in this review performed in Swedish and Norwegian populations reported a significant increase in NKp46 expression in patients compared with HC [48]. A repeated investigation by Rivas and colleagues in a Spanish cohort reported lack of significance in NKp46 expression on NK cells of ME/CFS patients compared with HC [51]. The latter, as already mentioned, included a high number of participants all fulfilling the CCC, thereby increasing the statistical power and its ability to conclusively detect notable differences between groups. The disparity in findings may be attributable to the use of different case definitions to identify ME/CFS patients and that these studies were completed in different countries. However, though observations were conflicting, changes in NK cell receptor profiles should not be discredited until additional research is completed.

NKG2C, a KIR activating receptor, was reported significantly reduced in ME/CFS patients compared with HC [51]. Conversely, Theorell and colleagues reported no differences in NKG2C [47]. Both papers recruited a large sample size fulfilling the CCC criteria. The discrepancies in findings may emphasise the immunological heterogeneity of ME/CFS. One paper examining multiple KIRs reported a significant increase in several KIRs [46]. Moreover, Rivas et al. reported that changes in NKG2C NK cell expression along with changes in regulatory T lymphocytes phenotypes had 70% accuracy when identifying cases of ME/CFS. Additional research is recommended on NK cell receptors as unbalanced inhibitory and activating receptors may contribute to impaired NK cell cytotoxicity in a subset of patients.

This systematic review included ten papers that examined NK cell cytotoxicity in ME/CFS patients compared with HC. Seven of these reported a significant decrease in NK cell cytotoxicity in ME/CFS. Brenu et al. was the first to report that NK cell cytotoxicity is consistently reduced over 12 months of illness [43]. The loss of overall NK cell cytotoxic activity is the most reliable report among all outcomes examined in this systematic review. Additionally, another investigation by Masuda and colleagues reported a significant reduction in NK cell cytotoxicity in ME/CFS patients compared with non-ME/CFS fatigued controls [18]. Note that this publication was excluded from this review due to the inclusion of chronic fatigue patients as a comparison group. Collectively, the papers included in this review demonstrate that reduced NK cell cytotoxicity is a useful indicator of immune dysfunction in ME/CFS patients. However, the evidence for why this reduction occurs is limited and requires additional research into possible ME/CFS subsets.

Degranulation is measured by surface expression of CD107a and CD107b [35]. Similar to other areas of NK cell research in ME/CFS patients, there are inconsistent reports of changes in degranulation compared with HC. One paper included in this review reported a significant increase in CD107a after stimulation using K562 cell line [25]. Huth et al. reported an increase in CD107a on CD56^Dim^ NK cells in ME/CFS patients compared with HC; however, this did not reach significance likely due to the small sample size [23]. An increase in NK cell degranulation in ME/CFS patients may lead to the inability to induce sufficient cytotoxicity resulting in increased activation. Additionally, due to abnormalities in NK cell receptors and MAPK phosphorylation, along with new evidence of impaired Ca^2+^ influx in NK cells, dysregulated cellular pathways may compromise degranulation in ME/CFS patients.

Changes in lytic proteins are not always consistent findings, but anomalies in perforin and granzyme B levels were reported [14, 21, 25]. There are many theories regarding the disparities observed in NK cells. Researchers suggest that it is a consequence of paucities in lytic proteins. Lytic proteins are vital for the immune response due to their involvement in elimination of pathogens as part of immune surveillance [59]. Perforin knockout mice had abnormal immune function and were at increased risk of infection, developing autoimmune diseases and lymphomas [60]. Theorell and colleagues in addition to measuring intracellular lytic proteins, investigated polymorphisms to *PRF1* [47]. While most ME/CFS participants were determined to have reduced perforin levels compared with HC, only one participant had the *PRF1* p.491 V variant that explained these low levels. Although, the mechanism responsible for reduced perforin in ME/CFS remains unknown, it is still believed to contribute to the loss of NK cell cytotoxicity in these patients.

Impaired phosphorylation of MAP kinases and p38 has been implicated in the pathogenesis of ME/CFS and other chronic inflammatory diseases [61]. MAPK phosphorylation mediates fundamental immunological processes leading to cytokine translation, polarisation of cytolytic granules and release of lytic proteins. Huth et al. reported an increased phosphorylation of MEK1/2 in CD56^Bright^ NK cells in ME/CFS patients [23], which may explain previously reported increased IFN-γ production [25]. Moreover, one paper included in this review reported decreased inhibition of IFN-γ production compared with HC after in vitro treatment with adrenaline [47], possibly indicating abnormalities in NK cell signalling of ME/CFS patients or impaired receptor function. Moreover, the significant reduction reported in ERK1/2 phosphorylation reported in ME/CFS may be positively correlated with impaired Ca^2+^ mobilisation described in recent publications by Nguyen and colleagues. NK cells are dependent on Ca^2+^ for the recruitment and phosphorylation of MAP kinases in addition to translation of lytic proteins, creation of the immune synapse, polarisation of cytolytic granules and the release of lytic proteins [62, 63].

Recent reports of impaired TRP ion channel function in ME/CFS patients may provide an explanation for immune dysfunction. Two papers included in this review examined TRPM3 channel expression and Ca^2+^ influx in addition to NK cell function [5, 49]. A significant reduction of TRPM3 surface expression was reported on NK cells in ME/CFS patients compared with HC along with a reduction in cytoplasmic Ca^2+^ in response to Ca^2+^ modulators. Moreover, a recent electrophysiology investigation used whole-cell patch clamp techniques to report impaired TRPM3 function in NK cells CFS patients/CFS patients and HC [64]. This paper was not included in this review as other NK cell features were not reported. There is growing evidence to suggest the underlying pathomechanism for ME/CFS involves ion channelopathy. As TRP channels are expressed ubiquitously across multiple organ systems, NK cells may act as a suitable model for other TRPM3 expressing tissues and to explore their functions.

### Quality assessment

Quality assessment was mostly consistent among several studies. While all papers were reported as not applicable or unclear for standardisation or reliability measurement of exposure, all publications used similar methods that are considered standard including flow cytometry and ^51^Cr release assay. Four of the included publications unfortunately did not provide details for defining their methods to correct for multiple comparisons during statistical analysis [2, 5, 44, 50]. Shortcomings were due to limited information on sources of confounding variables and sources of bias. Although there was no direct mention of controlling for confounding variables, studies attempted to address confounding in the following ways: (i) sex- and age-matching; and (ii) restricting comorbidities including but not limited to hypothyroidism et cetera. Selection bias may be a particular issue with some papers as limited information was provided regarding the recruitment of HC. Additional information should be provided regarding the clinical history of all participants including but not limited to ME/CFS onset, routine medications and comorbidities in addition to relevant medical information of HC. The selection criteria for ME/CFS patients appeared mostly consistent throughout all publications and all adhered to internationally accepted criteria. Conclusions drawn from all publications in this systematic review are consistent in many respects. For example, aberrations in NK cell cytotoxic activity or receptor profiles are significant immunological issues in ME/CFS patients that may compromise their ability to battle infections. Any shortcomings are unlikely to discredit the merit of the findings generated by these studies as they are not considered major limitations. However, it is recommended that for future investigations in this area, information be provided for patient socio-demographics, methods of participant recruitment and justification for the reported sample size.

## Conclusion

The aim of this systematic review was to examine the current literature available on NK cell cytotoxicity, cytokine production, lytic protein levels, degranulation and immunophenotypes in ME/CFS patients. Some of the publications included in this review provided evidence to suggest that NK cells may represent an important biological marker for investigating and identifying subsets of ME/CFS patients. NK cell cytotoxicity, and perforin levels to a lesser extent, remained a consistent immunological consequence of ME/CFS to provide a suitable foundation for future research in this area.

## Supplementary information

**Additional file 1.** Supplementary Table: The Joanna Briggs Institute Checklist for Case Control Studies and Downs and Black cheklist. Items 3, 4, 5, 8, 9, 13, 14, 15, 17-19, 21, 23-27 of the Downs and Black checklist were removed due to their specificity for intervention studies and overlap with the JBI checklist. *Abbreviations: JBI, Joanna Briggs Institute; Y, Yes; N, No; N/A not applicable; U, unclear.*

## Abbreviations

^51^Cr: Chromium
AChR: Acetylcholine receptors AChR
Ca^2+^: Calcium
CCC: Canadian Consensus Criteria
CFS: Chronic fatigue syndrome
DNA: Deoxyribonucleic acid
FM: Fibromyalgia
HC: Healthy controls
ICC: International Consensus Criteria
IFN-γ: Interferon gamma
IL: Interleukin
JBI: Joanna Briggs Institute
KIR: Killer-cell immunoglobulin-like receptors
KPS: Karnofsky Performance Scale
MAPK: Mitogen activation protein kinases
ME: Myalgic encephalomyelitis
MS: Multiple sclerosis
NCR: Natural cytotoxicity receptors
NK: Natural killer
NKG2D: NK receptor group 2 member D
PRISMA: Preferred Reporting Items for Systematic Reviews and Meta-Analyses
QoL: Quality of life
qRT-PCR: Qualitative reverse transcription polymerase chain reaction
SF-36: Short Form 36-item Health Survey
SLAM: Signalling lymphocytic activation molecules
SNP: Single-nucleotide polymorphisms
TNF-α: Tumour necrosis factor alpha
TRP: Transient receptor potential
TRPC: TRP canonical
TRPM: TRP melastatin
