# Supplementary material for: A systematic review of natural killer cells profile and cytotoxic function in myalgic encephalomyelitis/chronic fatigue syndrome
Source: Syst Rev. 2019 Nov 14;8:279. doi: 10.1186/s13643-019-1202-6 (PMC6857215; doi:10.1186/s13643-019-1202-6)
Supplement: Supplementary file 1 — Additional file 1. Supplementary Table: The Joanna Briggs Institute Checklist for Case Control Studies and Downs and Black cheklist. Items 3, 4, 5, 8, 9, 13, 14, 15, 17-19, 21, 23-27 of the Downs and Black checklist were removed due to their specificity for intervention studies and overlap with the JBI checklist. Abbreviations: JBI, Joanna Briggs Institute; Y, Yes; N, No; N/A not applicable; U, unclear. [file 13643_2019_1202_MOESM1_ESM.docx]

|  |  | **CHECKLIST ITEMS** | | | | | | | | | | | | | | | | | | | | |
| --- | --- | --- | --- | --- | --- | --- | --- | --- | --- | --- | --- | --- | --- | --- | --- | --- | --- | --- | --- | --- | --- | --- |
|  |  | **JBI Checklist** | | | | | | | | | | **Downs and Black Checklist** | | | | | | | | | | |
| **Author** | **Year** | **1** | **2** | **3** | **4** | **5** | **6** | **7** | **8** | **9** | **10** | **1** | **2** | **6** | **7** | **10** | **11** | **12** | **16** | **20** | **22** |  |
| Brenu *et al.* | 2010 | Y | Y | Y | N/A | N/A | N | N | Y | N/A | Y | Y | Y | Y | Y | N | Y | N | Y | Y | U |  |
| Brenu *et al.* | 2011 | N | Y | Y | N/A | N/A | Y | Y | Y | N/A | N | Y | Y | Y | Y | N | Y | N | Y | Y | U |  |
| Brenu *et al.* | 2012 | N | Y | Y | N/A | N/A | Y | Y | Y | N/A | Y | Y | Y | Y | Y | N | Y | N | Y | Y | Y |  |
| Brenu *et al.* | 2013 | N | N | Y | N/A | N/A | Y | Y | Y | N/A | Y | Y | Y | Y | Y | Y | N | N | Y | Y | U |  |
| Curriu *et al.* | 2013 | N | Y | Y | N/A | N/A | Y | Y | Y | N/A | Y | Y | Y | Y | Y | Y | Y | N | Y | Y | U |  |
| Fletcher *et al.* | 2010 | N | Y | Y | N/A | N/A | Y | Y | Y | N/A | Y | Y | Y | Y | Y | Y | Y | N | Y | Y | U |  |
| Hardcastle *et al.* | 2015a | Y | Y | Y | N/A | N/A | Y | Y | Y | N/A | Y | Y | Y | Y | Y | Y | Y | N | Y | Y | U |  |
| Hardcastle *et al.* | 2015b | Y | Y | Y | N/A | N/A | Y | Y | Y | N/A | Y | Y | Y | Y | Y | Y | Y | N | Y | Y | U |  |
| Huth *et al.* | 2014 | N | N | Y | N/A | N/A | N | N | Y | N/A | Y | Y | Y | Y | Y | N | Y | N | Y | Y | U |  |
| Huth *et al.* | 2016 | N | Y | Y | U | U | Y | Y | Y | U | Y | Y | Y | Y | Y | N | N | N | Y | Y | U |  |
| Maher *et al.* | 2005 | N | N | Y | N/A | N/A | N | N | Y | N/A | Y | Y | Y | Y | Y | Y | N | N | Y | Y | U |  |
| Marshall-Gradisnik *et al.* | 2016 | N | N | Y | N/A | N/A | Y | Y | Y | N/A | N | Y | Y | Y | Y | N | N | N | Y | Y | U |  |
| Nguyen *et al.* | 2016 | Y | U | Y | N/A | N/A | Y | Y | Y | N/A | Y | Y | Y | Y | Y | N | N | N | Y | Y | U |  |
| Nguyen *et al.* | 2017 | N | Y | Y | U | U | Y | Y | Y | U | N | Y | Y | Y | Y | Y | Y | N | Y | Y | U |  |
| Rivas *et al.* | 2018 | Y | Y | Y | N/A | N/A | Y | Y | Y | N/A | Y | Y | Y | Y | Y | Y | Y | N | Y | Y | U |  |
| Stewart *et al.* | 2003 | Y | Y | Y | N/A | N/A | Y | Y | Y | N/A | N | Y | Y | Y | Y | Y | Y | N | Y | Y | U |  |
| Theorell *et al.* | 2017 | Y | Y | Y | N/A | N/A | N | N | Y | N/A | Y | Y | Y | Y | Y | Y | Y | N | Y | Y | U |  |

**Supplementary Table:** The Joanna Briggs Institute Checklist for Case Control Studies and Downs and Black checklist. Items 3, 4, 5, 8, 9, 13, 14, 15, 17-19, 21, 23-27 of the Downs and Black Checklist were removed due to their specificity for intervention studies and overlap with the JBI checklist. *Abbreviations: JBI, Joanna Briggs Institute; Y, Yes; N, No; N/A, not applicable; U, unclear.*

JBI Checklist items:
1. Were the groups comparable other than the presence of disease in cases or the absence of disease in controls?
2. Were cases and controls matched appropriately?
3. Were the same criteria used for identification of cases and controls?
4. Was exposure measured in a standard, valid and reliable way?
5. Was exposure measured in the same way for cases and controls?
6. Were confounding factors identified?
7. Were strategies to deal with confounding factors stated?
8. Were outcomes assessed in a standard, valid and reliable way for cases and controls?
9. Was the exposure period of interest long enough to be meaningful?
10. Was appropriate statistical analysis used?

Downs and Black Checklist Items:

1. Is the hypothesis/aim/objective of the study clearly described?
2. Are the main outcomes to be measured clearly described in the Introduction or Methods section?
3. Are the characteristics of the patients included in the study clearly described?
4. Are the interventions of interest clearly described?
5. Are the distributions of principal confounders in each group of subjects to be compared clearly described?
6. Are the main findings of the study clearly described?
7. Does the study provide estimates of the random variability in the data for the main outcomes?
8. Have all important adverse events that may be a consequence of the intervention been reported?
9. Have the characteristics of patients lost to follow-up been described?
10. Have actual probability values been reported (e.g. 0.035 rather than <0.05) for the main outcomes except where the probability value is less than 0.001?
11. Where the subjects asked to participate in the study representative of the entire population from which they were recruited?
12. Were those subjects who were prepared to participate representative of the entire population from which they were recruited?
13. Were the staff, places and facilities where the patients were treated, representative of the treatment the majority of patients receive?
14. Was an attempt made to blind study subjects to the intervention they received?
15. Was an attempt made to blind those measuring the main outcomes of the intervention?
16. If any of the results of the study were based on “data dredging”, was this made clear?
17. In trials and cohort studies, do the analyses adjust for different lengths of follow-up of patients, or in case-control studies, is the time period between the intervention and outcome the same for cases and controls?
18. Were the statistical tests used to assess the main outcomes appropriate?
19. Was compliance with the intervention/s reliable?
20. Were the main outcome measures used accurate (valid and reliable)?
21. Were the patients in different intervention groups (trial and cohort studies) or were the cases and controls (case-control studies) recruited from the same population?
22. Were study subjects in different intervention groups (trials and cohort studies) or were the cases and controls (case-control studies) recruited over the same period of time?
23. Were study subjects randomised to intervention groups?
24. Was the randomised intervention assignment concealed from both patients and health care staff until recruitment was complete and irrevocable?
25. Was there adequate adjustment for confounding in the analyses from which the main findings were drawn?
26. Were losses of patients to follow-up taken into account?
27. Did the study have sufficient power to detect a clinically important effect where the probability value for a difference being due to chance is less than 5%?

Justification

Brenu *et al.* 2010

JBI Checklist

1. Participants age- and sex-matched
2. CFS patients sourced from community-based sample in NSW and QLD; HC sourced from local community area
3. CFS patients chosen after completion of questionnaire adapted from CDC 1994 case definition and duration of illness was more than 5 years. Controls didn’t fulfil these criteria
4. Exposure not part of study as it is a comparison of lymphocyte, NK, neutrophil, erythrocyte, and fibrinogen features
5. As above
6. Confounders not provided or analysed
7. As above
8. Used flow cytometry and other standardised kits to measure endpoints
9. No exposure
10. Used independent sample t-test: assumes normal distribution in each group and equal variances for the two groups, assumes each case represents a different person (true)

Downs and Black

1. Aim and objective clearly described in abstract and introduction

2. The main outcomes to be measured were described in both introduction and methods sections

6. The main findings are described in the results section

7. Standard error, standard deviation and/or confidence intervals were reported

10. Probability values were reported as <0.05.

11. ME/CFS patients sourced from community-based sample in NSW and QLD; HC sourced from local community area

12.Validation that participants were representative of entire population from which they were recruited not given

16. No retrospective unplanned analyses were reported

20. Outcomes measured clearly described

22. Unable to determine as paper does not specify time period

Brenu *et al.* 2011

JBI Checklist

1. Doesn’t list how cases and controls were matched, just population characteristics
2. All participants were recruited from QLD and NSW through CFS/ME support groups, newspaper and email advertisements
3. CFS patients chosen after completion of questionnaire adapted from CDC 1994 case definition and duration of illness was more than 5 years. Controls didn’t fulfil these criteria
4. Exposure not part of study as it is a comparison of lymphocyte, NK, neutrophil, erythrocyte, and fibrinogen features
5. As above
6. Confounders provided as exclusion criteria – autoimmune disorders, psychosis, epilepsy, heart disease, pregnant/breastfeeding
7. Controlled for by exclusion (study design)
8. Used flow cytometry and other standardised kits to measure endpoints
9. No exposure
10. Used ANOVA and independent samples t-test. ANOVA assumes experimental errors of data are normally distributed, equal variance between groups, independence of samples, which are satisfied. However, authors did not report attempts to correct for multiple comparisons.

Downs and Black Checklist

1. Aim and objective clearly described in abstract and introduction

2. The main outcomes to be measured were described in both introduction and methods sections

6. The main findings are described in the results section

7. Standard error, standard deviation and/or confidence intervals were reported

10. Probability values were reported as <0.05.

11. All participants were recruited from QLD and NSW through CFS/ME support groups

12. Validation that participants were representative of entire population from which they were recruited not given

16. No retrospective unplanned analyses were reported

20. Outcomes measured clearly described

22. Unable to determine as paper does not specify time period

Brenu *et al.* 2012

JBI Checklist

1. Doesn’t list how cases and controls were matched, just population characteristics
2. CFS/ME patients and non-fatigued controls were recruited from an existing cohort in QLD and NSW
3. CFS/ME cohort met the 1994 CDC criteria, control group consisted of non-fatigued volunteers
4. Exposure not part of study; investigated NK cytotoxic activity, NK subsets, and T cell specific cytokine distribution
5. As above
6. Confounders provided as exclusion criteria – autoimmune disorders, psychosis, epilepsy, heart disease, pregnant/breastfeeding
7. Controlled for by exclusion (study design)
8. Used flow cytometry and other standardised kits to quantify outcomes
9. No exposure
10. Data analysed with ANOVA and repeated measures, corrections applied where sphericity assumption breached (repeated measures ANOVA, variances of the differences between all combinations of the conditions are equal), Bonferroni post-hoc analysis performed

Downs and Black Checklist

1. Aim and objective clearly described in abstract and introduction

2. The main outcomes to be measured were described in both introduction and methods sections

6. The main findings are described in the results section

7. Standard error, standard deviation and/or confidence intervals were reported

10. Probability values were reported as <0.05.

11. CFS/ME patients and non-fatigued controls were recruited from an existing cohort in QLD and NSW

12. Validation that participants were representative of entire population from which they were recruited not given

16. No retrospective unplanned analyses were reported

20. Outcomes measured clearly described

22. Recruitment and participant scheduling was performed over the same time period.

Brenu *et al.* 2013

JBI Checklist

1. Doesn’t list how cases and controls were matched, just mean age of subjects
2. Doesn’t state source population
3. CFS/ME cohort met the 1994 CDC criteria, non-fatigued controls
4. Exposure not part of the study, assess cell phenotype, surface antigens, adhesion molecules, receptors, intracellular functional proteins, NK cell cytotoxic activity, degranulation, IFN-γ, and Th1/2/17 cytokines
5. As above
6. Confounders provided as exclusion criteria – autoimmune disorders, psychosis, epilepsy, cardiac related disorders, pregnant or breastfeeding
7. Controlled for by exclusion (study design)
8. Used flow cytometry for all experiments
9. No exposure
10. Pairwise comparison using multivariate testing used to perform comparative assessments of all data generated from all participants, analysis of variance and independent sample t-test used to determine significance, Bonferroni for post-hoc analysis, normality tested appropriately, Spearman’s rank correlation for non-parametric testing of correlations

Downs and Black Checklist

1. Aim and objective clearly described in abstract and introduction

2. The main outcomes to be measured were described in both introduction and methods sections

6. The main findings are described in the results section

7. Standard error, standard deviation and/or confidence intervals were reported

10. Actual probability values were given.

11. Study did not identify source population

12. Validation that participants were representative of entire population from which they were recruited not given

16. No retrospective unplanned analyses were reported

20. Outcomes measured clearly described

22. Unable to determine as paper does not specify time period

Curriu *et al.* 2013

JBI Checklist

1. Doesn’t list how cases and controls were matched, just population characteristics
2. CFS/ME patients selected from cohorts of CFS clinical units (CFS unit, Tarragona, Span and Vall d’Hebron University Hospital, Barcelona, Spain), control recruitment method not stated
3. CFS/ME cases fulfilled the 1994 Fukuda criteria and confirmed diagnosis of more than 2 years, all eligible subjects over age 18 and absence of current illness
4. No exposure
5. As above
6. Confounding factors listed as exclusion criteria – diabetes, hypertension, chronic obstructive pulmonary disease, inflammatory bowel or Crohn’s disease, rheumatoid arthritis, Parkinson or Huntington disorders, schizophrenia, organic mental disorders, substance use disorders, multiple sclerosis, and BMI > 30 kg/m^2^
7. Controlled for by exclusion (study design)
8. Used flow cytometry for all endpoints
9. No exposure
10. Continuous variables expressed as median (inter-quartile range) and compared using the Mann-Whitney non-parametric test, chi-square or Fisher-exact test used to evaluate discrete variables expressed as percentages

Downs and Black Checklist

1. Aim and objective clearly described in abstract and introduction

2. The main outcomes to be measured were described in both introduction and methods sections

6. The main findings are described in the results section

7. Standard error, standard deviation and/or confidence intervals were reported

10. Actual probability values were given.

11. CFS/ME patients selected from cohorts of CFS clinical units

12. Validation that participants were representative of entire population from which they were recruited not given

16. No retrospective unplanned analyses were reported

20. Outcomes measured clearly described

22. Unable to determine as paper does not specify time period

Fletcher *et al.* 2010

JBI Checklist

1. Doesn’t list how cases and controls were matched, just mean age and %female
2. CFS/ME patients were in research studies (NIH, Chronic fatigue and immunodeficiency syndrome [CFIDS] association or University of Miami), healthy controls from University of Griffith, NIH, or CFIDS
3. CFS/ME fulfilled the CDC clinical diagnostic criteria, controls completed a medical and psychiatric history
4. No exposure
5. As above
6. Confounding factors listed as exclusion criteria, CFS – any active medical condition that could explain the presence of chronic fatigue including diabetes, current use of immune-modulatory or antibiotic medications, past or present psychiatric diagnosis of psychosis, dementia, major depressive disorder, anorexia or bulimia nervosa, alcohol/substance abuse within two years of the onset of the fatigue or anytime thereafter. Controls – active medical or psychiatric conditions, immunomodulating medications, alcohol/substance abuse
7. Controlled for by exclusion (study design)
8. NK cytotoxicity assessed using chromium release assay (common and validated method), flow cytometry used to determine lymphocyte subsets and cell surface protein concentrations from whole blood, soluble CD26 assayed using ELISA kit
9. No exposure
10. Nonparametric Mann-Whitney test used to determine magnitude of between group differences, nonparametric Spearman test used to determine correlations, diagnostic accuracy of markers assessed using nonparametric receiver operating characteristics for true positive and true negative occurrences (uses all of the data, makes no parametric assumptions, provides unbiased estimates of sensitivity and specificity

Downs and Black Checklist

1. Aim and objective clearly described in abstract and introduction

2. The main outcomes to be measured were described in both introduction and methods sections

6. The main findings are described in the results section

7. Standard error, standard deviation and/or confidence intervals were reported

10. Actual probability values were given.

11. ME/CFS participants selected from University of Miami Miller School of Medicine CFS and Immunodeficiency Clinic after diagnosis.

12. Validation that participants were representative of entire population from which they were recruited not given

16. No retrospective unplanned analyses were reported

20. Outcomes measured clearly described

22. Unable to determine as paper does not specify time period

Hardcastle *et al.* 2015a

JBI Checklist

1. CFS/ME patients and non-fatigued controls were age- and sex-matched
2. Participants from QLD and NSW were again approached for this follow-up study
3. CFS/ME patients fulfilled the 1994 Fukuda criteria, had the illness for at least 6 months prior to the study, and sorted as moderate or severe using a questionnaire to assess symptomatology, health status, QoL, severity, and mobility. Healthy controls did not fulfil these criteria
4. No exposure
5. As above
6. Confounding factors provided as exclusion criteria, CFS – previous autoimmune disorder, psychosis, heart disease, thyroid-related disorders, pregnant/breastfeeding, smoking, or experiencing symptoms of CFS/ME that didn’t conform to the Fukuda criteria
7. Controlled for by exclusion (study design)
8. Outcomes assessed using flow cytometry
9. No exposure
10. Paired t-tests were used to examine changes in each immune parameter between 0 and 6 months for each of the groups, ANOVA used to assess the interaction of time and group for 6-month single time point analysis if normally distributed and Shapiro-Wilk and Kruskal-Wallis test used if not normally distributed, Bonferroni and Mann-Whitney post-hoc tests used to determine significance for parametric and non-parametric tests, respectively, extreme outliers eliminated from analysis

Downs and Black Checklist

1. Aim and objective clearly described in abstract and introduction

2. The main outcomes to be measured were described in both introduction and methods sections

6. The main findings are described in the results section

7. Standard error, standard deviation and/or confidence intervals were reported

10. Actual probability values were given.

11. Participants were recruited from Queensland and New South Wales in Australia.

12. Validation that participants were representative of entire population from which they were recruited not given

16. No retrospective unplanned analyses were reported

20. Outcomes measured clearly described

22. Unable to determine as paper does not specify time period

Hardcastle *et al.* 2015b

JBI Checklist

1. All participants age- and sex-matched
2. Participants recruited from QLD and NSW through CFS/ME support groups, email advertisements, and social media
3. CFS/ME patients fulfilled 1994 Fukuda criteria and had illness for at least 6 months prior to the study, all had been diagnosed by a primary care physician
4. No exposure
5. As above
6. Confounding factors listed as exclusion criteria – previously diagnosed with autoimmune disorder, multiple sclerosis, psychosis, major depression, heart disease, thyroid-related disorders, pregnant/breastfeeding, smoker, or experiencing symptoms of CFS/ME that didn’t conform to 1994 Fukuda definition. Data also collected and analysed regarding onset of illness, presence, frequency, and severity of symptoms, comorbidities, overall health, and QoL
7. Controlled for by exclusion (study design) and additional data collected to allow participants to be analysed on an individual basis to minimise confounding
8. Outcomes assessed using flow cytometry and other validated kits
9. No exposure
10. ANOVA used for normally distributed data, otherwise Kruskal-Wallis test of independent variables based on rank sums to determine magnitude of group differences was performed. Bonferroni post-hoc or Mann-Whitney post-hic was performed for parametric and nonparametric data, respectively. Extreme outliers identified using box plot and eliminated from analysis

Downs and Black Checklist

1. Aim and objective clearly described in abstract and introduction

2. The main outcomes to be measured were described in both introduction and methods sections

6. The main findings are described in the results section

7. Standard error, standard deviation and/or confidence intervals were reported

10. Actual probability values were given.

11. Participants were recruited from Queensland and New South Wales in Australia.

12. Validation that participants were representative of entire population from which they were recruited not given

16. No retrospective unplanned analyses were reported

20. Outcomes measured clearly described

22. Unable to determine as paper does not specify time period

Huth *et al.* 2014

JBI Checklist

1. Doesn’t detail how patients and controls were matched
2. Doesn’t mention what the source population for patients or controls was
3. CFS/ME patients fulfilled the 1994 Fukuda criteria (identified through an online questionnaire) and healthy controls were not fatigued
4. No exposure
5. As above
6. No confounders identified
7. As above
8. All outcomes assessed using flow cytometry
9. No exposure
10. Shapiro-Wilk test used to test for Gaussian distribution (normal distribution), nonparametric Mann-Whitney test used to identify significance between CFS/ME and HC for NK parameters. Two-way ANOVA (interaction between two independent variables on the dependent variable) with Tukey’s multiple comparisons test (compares the difference between each pair of means like a t-test but appropriately adjusts for multiple testing) used to identify significant differences in NK phenotype expression

Downs and Black Checklist

1. Aim and objective clearly described in abstract and introduction

2. The main outcomes to be measured were described in both introduction and methods sections

6. The main findings are described in the results section

7. Standard error, standard deviation and/or confidence intervals were reported

10. Probability values were reported as <0.05.

11.

12. Validation that participants were representative of entire population from which they were recruited not given

16. No retrospective unplanned analyses were reported

20. Outcomes measured clearly described

22. Unable to determine as paper does not specify time period

Huth *et al.* 2016

JBI Checklist

1. Doesn’t describe how patients and controls were matched
2. Source population for CFS/ME patients and non-fatigued controls was the NCNED participant database
3. Participants completed online questionnaire based on 1994 Fukuda definition, controls were non-fatigued
4. No exposure (can argue that stimulation of NK cells is an exposure and how each group responds to this stimulation is compared)
5. As above
6. Listed epilepsy, thyroid conditions, psychosis, diabetes, cardiac disorders, smoking, pregnant/breastfeeding, immunological and inflammatory diseases as exclusionary criteria for confounding
7. Controlled for by exclusion (study design)
8. Endpoints measured using flow cytometry
9. No exposure
10. Mann-Whitney U test (nonparametric alternative to independent samples t-test) used to determine significant differences between CFS/ME and controls. Kruskal-Wallis multiple comparisons test (used to compare two or more independent samples of equal or different sample sizes, nonparametric) used to identify significant differences in NK parameters before and after stimulation (with K562 or phorbol-12-myristate-13-acetate plus ionomycin)

Downs and Black Checklist

1. Aim and objective clearly described in abstract and introduction

2. The main outcomes to be measured were described in both introduction and methods sections

6. The main findings are described in the results section

7. Standard error, standard deviation and/or confidence intervals were reported

10. Probability values were reported as <0.05.

11. Study did not identify source population

12. Validation that participants were representative of entire population from which they were recruited not given

16. No retrospective unplanned analyses were reported

20. Outcomes measured clearly described

22. Unable to determine as paper does not specify time period

Maher *et al.* 2005

JBI Checklist

1. Doesn’t describe how patients and controls were matched
2. Doesn’t identify the source population of patients or controls
3. CFS/ME patients met the CDC 1994 case definition, healthy controls were apparently healthy but sedentary
4. No exposure
5. As above
6. No confounders identified
7. As above
8. Flow cytometry used to determine endpoints
9. No exposure
10. All analyses were performed using the Student’s t-test and correlations made using the Pearson product-moment test

Downs and Black Checklist

1. Aim and objective clearly described in abstract and introduction

2. The main outcomes to be measured were described in both introduction and methods sections

6. The main findings are described in the results section

7. Standard error, standard deviation and/or confidence intervals were reported

10. Actual probability values were given.

11. Study did not identify source population

12. Validation that participants were representative of entire population from which they were recruited not given

16. No retrospective unplanned analyses were reported

20. Outcomes measured clearly described

22. Unable to determine as paper does not specify time period

Marshall-Gradisnik *et al.* 2016

JBI Checklist

1. Method of matching not reported, provides demographic data
2. Doesn’t identify the source population
3. CFS/ME patients identified met the 1994 CDC criteria, healthy controls reported no medical history, symptoms of prolonged fatigue, or illness of any kind
4. No exposure
5. As above
6. Confounders not provided but does only include controls that have reported no medical history, symptoms of prolonged fatigue, illness of any kind, and have been screened using routine pathology
7. Controlled for by exclusion (study design)
8. Flow cytometry, nanodrop, and microarray were used to measure outcomes
9. No exposure
10. ANOVA used to compare CFS/ME with healthy controls for different parameters, two-column chi-squared test was used to examine differences in ME/CFS patients and healthy controls for genomic analysis. However, authors did not report attempts to correct for multiple comparisons.

Downs and Black Checklist

1. Aim and objective clearly described in abstract and introduction

2. The main outcomes to be measured were described in both introduction and methods sections

6. The main findings are described in the results section

7. Standard error, standard deviation and/or confidence intervals were reported

10. Probability values were reported as <0.05.

11. Study did not identify source population

12. Validation that participants were representative of entire population from which they were recruited not given

16. No retrospective unplanned analyses were reported

20. Outcomes measured clearly described

22. Unable to determine as paper does not specify time period

Nguyen *et al.* 2016

JBI Checklist

1. Patients and controlled were age-matched (not sex-matched)
2. Reports participants all as residents of Australia at the time of collection but not the source population
3. CFS/ME patients defined according to the Fukuda criteria, healthy controls did not meet any CFS/ME criteria
4. No exposure
5. As above
6. Exclusion criteria included smokers, breastfeeding/pregnant
7. Controlled for by exclusion (study design)
8. Outcomes assessed using flow cytometry
9. No exposure
10. Data was analysed by MANOVA and post-hoc testing was used to determine if these differences were significant. Levene test was used to analyse homogeneity of variance between groups

Downs and Black Checklist

1. Aim and objective clearly described in abstract and introduction

2. The main outcomes to be measured were described in both introduction and methods sections

6. The main findings are described in the results section

7. Standard error, standard deviation and/or confidence intervals were reported

10. Probability values were reported as <0.05.

11. Study did not identify source population

12. Validation that participants were representative of entire population from which they were recruited not given

16. No retrospective unplanned analyses were reported

20. Outcomes measured clearly described

22. Unable to determine as paper does not specify time period

Nguyen *et al.* 2017

JBI Checklist

1. Doesn’t detail how patients were matched
2. Identifies the source population for participants as south east Queensland region
3. CFS/ME patients meeting the Fukuda criteria were included in this study, healthy controls did not meet these criteria
4. NK cells were treated with ionomycin, 2-APB, thapsigargin, and PregS. Compared cytotoxic activity following treatment with drugs
5. Above measured in the same way for cases and controls (flow cytometry) by apoptotic marker expression
6. Exclusion criteria: previous history of smoking or chronic disease (autoimmune diseases, cardiac diseases, primary psychological disorders), pregnant/breastfeeding, taking hormone replacement therapy and immunoregulatory medications
7. Controlled for by exclusion (study design)
8. Outcomes assessed by flow cytometry
9. Exposure was instantaneous drug treatment and measurement of ion currents
10. Data was analysed by MANOVA comparing CFS/ME with HC as well as within each group. Mann-Whitney U test was used to determine specific relationship tests between each intervention on TRPM3, CD69, and CD107a surface expression for NK cell subsets. However, authors did not report attempts to correct for multiple comparisons.

Downs and Black Checklist

1. Aim and objective clearly described in abstract and introduction

2. The main outcomes to be measured were described in both introduction and methods sections

6. The main findings are described in the results section

7. Standard error, standard deviation and/or confidence intervals were reported

10. Actual probability values were given.

11. Study identified source population to be from same area and used a local recruitment database.

12. Validation that participants were representative of entire population from which they were recruited not given

16. No retrospective unplanned analyses were reported

20. Outcomes measured clearly described

22. Unable to determine as paper does not specify time period

Rivas *et al.* 2018

JBI Checklist

1. Participants were matched for age and gender
2. Patients were sourced from those diagnosed with ME/CFS in Barcelona, Madrid, and San Sebastian
3. ME/CFS patients met the revised CCC criteria 2010 assessed by two medical practitioners and following a questionnaire, healthy controls did not meet these criteria and couldn’t be a first- or second-degree relative of recruited patients
4. No exposure
5. As above
6. Exclusion criteria, ME/CFS – didn’t meet the revised CCC 2010 criteria, had medical condition that could justify the symptoms of the disease. Controls – first- or second-degree relative of the recruited patients
7. Controlled for by exclusion (study design)
8. Symptom severity assessed with self-reported SF-36 questionnaire, level of fatigue by Scale of Degree of Impairment. Other outcomes were measured using flow cytometry, chemiluminescence, and enzyme immunoassay
9. No exposure
10. Differential cell populations between patients and controls were measured using Mann-Whitney test followed by multiple correction, correlation was determined using Spearman’s rho

Downs and Black Checklist

1. Aim and objective clearly described in abstract and introduction

2. The main outcomes to be measured were described in both introduction and methods sections

6. The main findings are described in the results section

7. Standard error, standard deviation and/or confidence intervals were reported

10. Actual probability values were given.

11. Patients were selected from the same geographical area of Barcelona, Madrid and San Sebastian.

12. Validation that participants were representative of entire population from which they were recruited not given

16. No retrospective unplanned analyses were reported

20. Outcomes measured clearly described

22. Unable to determine as paper does not specify time period

Stewart *et al.* 2003

JBI Checklist

1. CFS/ME patients were matched by age, sex, and geographical location
2. Participants were recruited from upstate New York (Lyndonville and Buffalo), Lyndonville controls from same clinical practice as patients, Buffalo controls from staff members at Roswell Park Cancer Institute and the University of Buffalo and graduate students at the same locations
3. CFS/ME patients met 1994 CDC criteria and 1988 CFS criteria, underwent comprehensive diagnostic clinical and laboratory evaluation. Controls interviewed regarding their general health and the health of the other individuals in their household
4. No exposure
5. As above
6. Controls were excluded if they had a diagnosed history of chronic disease, reported they had experienced CFS-like symptoms on an intermittent basis but had never consulted a physician or had CFS-like symptoms persisting at least 6 months, or had lived in the same household as someone with CFS or another control in this study. Unclear for CFS patients the exclusion criteria
7. Controlled for by exclusion (study design)
8. Samples analysed with flow cytometry
9. No exposure
10. Cohorts compared using t-test or Mann-Whitney U test as appropriate, ANOVA or Kruskal-Wallis test used to simultaneously compare for each of the four NK cell subsets of interest. However, authors did not report attempts to correct for multiple comparisons.

Downs and Black Checklist

1. Aim and objective clearly described in abstract and introduction

2. The main outcomes to be measured were described in both introduction and methods sections

6. The main findings are described in the results section

7. Standard error, standard deviation and/or confidence intervals were reported

10. Actual probability values were given.

11. Participants were recruited from specific areas of New York. Cases were matched not only be age and sex, but also geographic location.

12. Validation that participants were representative of entire population from which they were recruited not given

16. No retrospective unplanned analyses were reported

20. Outcomes measured clearly described

22. Unable to determine as paper does not specify time period

Theorell *et al.* 2017

JBI Checklist

1. CFS/ME patients and controls were age- and sex-matched
2. Swedish patients were recruited at the ME/CFS rehabilitation unit, Danderyd University Hospital, Norwegian patients were recruited at the CFS/ME centre, Oslo university hospital. Healthy controls recruited from Oslo University Hospital blood bank and Stockholm blood bank
3. CFS/ME patients met the CCC criteria, healthy controls didn’t
4. No exposure
5. As above
6. Confounders not identified
7. As above
8. Outcomes were measured using flow cytometry, genetic analyses (TaqMan genotyping assay according to manufacturer instructions)
9. No exposure
10. Supplementary material accessed, analyses are appropriate

Downs and Black Checklist

1. Aim and objective clearly described in abstract and introduction

2. The main outcomes to be measured were described in both introduction and methods sections

6. The main findings are described in the results section

7. Standard error, standard deviation and/or confidence intervals were reported

10. Actual probability values were given.

11. Participants were recruited from a specified rehabilitation unit in same geographic area.

12. Validation that participants were representative of entire population from which they were recruited not given

16. No retrospective unplanned analyses were reported

20. Outcomes measured clearly described

22. Unable to determine as paper does not specify time period
